# Integrating pulmonary embolism response teams: perspectives and outcomes from a European and German survey analysis

**DOI:** 10.1186/s12959-026-00872-7

**Published:** 2026-08-04

**Authors:** Lukas Hobohm, Karsten Stannek, Karsten Keller, Ioannis T. Farmakis, Tobias Tichelbaecker, Philipp Lurz, Christian Perings, Stavros Konstantinides, Ingo Ahrens

**Affiliations:** 1 Department of Cardiology, University Medical Center Mainz (Johannes Gutenberg- University Mainz), City Mainz, Germany; 2 Center for Thrombosis and Hemostasis (CTH), University Medical Center Mainz (Johannes Gutenberg-University Mainz), City Mainz, Germany; 3 Medizinische Klinik und Poliklinik I, University Hospital, LMU Munich, City Munich, Germany; 4 Clinic III for Internal Medicine, Heart Centre of University Hospital of Cologne, City Cologne, Germany; 5 Department of Cardiology, Katholisches Klinikum Luenen, City Luenen, Germany; 6 Department of Cardiology and Medical Intensive Care, Augustinerinnen Hospital, Academic Teaching Hospital University of Cologne, City Cologne, Germany; 7 Medical Faculty, University of Freiburg, City Freiburg, Germany

**Keywords:** Pulmonary embolism, Team, PERT

## Abstract

In recent years, establishing multidisciplinary pulmonary embolism response teams (PERTs/EXPERT-PE) has become more prevalent. A survey was conducted to assess the availability and structure of PERTs in German and European hospitals. A survey was circulated among German hospital centers with cardiovascular expertise to obtain data on their current PE treatment approaches, the existence and features of PERTs, their activation methods, and their structural organization. Additionally, data regarding PE treatment priority relative to acute coronary syndrome and the availability of outpatient PE follow-up programs was gathered. Results were compared with those of European PE reference centers. Overall, 65 hospitals participated at this German survey with most PERTs available at university hospitals (63%), especially in West (63%) and Southern (62%). Although 61.5% of the German centers handle over 40 PE cases annually, only 52.3% of those hospitals already implemented a PERT, which is low in comparison to a European Survey among PE reference centers (80.8%). In 86% cases, PERT is led by Cardiology, followed by Angiology (6.9%), Pulmonology (4.6%), and Critical Care Medicine (2.3%). Regarding advanced treatment the majority of hospitals performed catheter-directed thrombolysis (72.2%) and catheter-based thrombectomy/aspiration (74.1%). A follow-up program for patients with PE is considerable higher in European reference centers opposed to German hospitals. This survey demonstrated that among large German cardiovascular centers PERTs are markedly less often present compared to European PE reference centers. Additionally, in German centers, PE follow-up programs are not widely implemented. This emphasizes the pressing need for awareness campaigns for acute and chronic PE.

## Introduction

Pulmonary embolism (PE) remains one of the most common and potentially life-threatening cardiovascular emergencies worldwide [1]. While anticoagulation represents the cornerstone of therapy, the management of high-risk and selected intermediate-risk cases often requires complex decision-making regarding advanced reperfusion strategies, including systemic thrombolysis, catheter-directed interventions, or surgical embolectomy [2]. In recent years, Pulmonary Embolism Response Teams (PERT) have been introduced in several healthcare systems to address these challenges [3]. By bringing together specialists from cardiology, pulmonology, radiology, intensive care, and cardiac surgery, PERTs aim to facilitate rapid interdisciplinary consultation and treatment decision in order to improve outcomes through individualized, evidence-based treatment pathways. Despite growing international interest, the structure, availability, and utilization of PERTs vary considerably between hospitals, regions, and healthcare systems, comprehensive insights into their role within the German healthcare system have been limited.

Therefore, the present survey was designed to capture the current state of PE management in Germany and to compare these findings with practices in established European pulmonary embolism centers.

## Methods

A nationwide survey was conducted to evaluate the availability, implementation, and organizational structure of PERTs in German hospitals. The questionnaire was distributed via E-mail to German hospital centers with established expertise in cardiovascular care, including tertiary care hospitals and specialized treatment centers (Arbeitsgemeinschaft Leitende kardiologische Krankenhausärzte e.V.). A total of 107 centers were contacted, of whom 65 centres replied. Respondents were asked to provide information on their current management strategies for acute PE, with a particular focus on the existence of a PERT, its composition, activation processes, and operational framework.

In addition, the survey addressed related aspects, such as the prioritization of PE management in comparison to acute coronary syndromes, as well as the availability and organization of structured outpatient follow-up programs for patients with PE. The collected data were analyzed to identify patterns in clinical practice and potential gaps in care delivery across different hospital settings.

To place the findings into a broader international context, results from German centers were compared with data among designated PE reference centers. PE reference centers were defined as European centers participating in major randomized controlled trials on PE. This comparison aimed to highlight similarities and differences in the structural and organizational approaches to PE care, with particular attention to the role and utilization of PERTs.

## Results

**Fig. 1:**
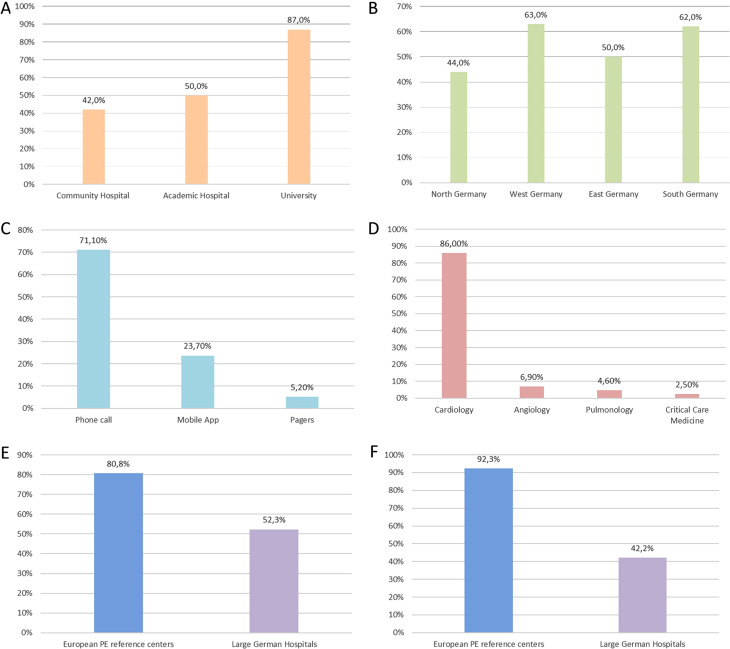
(**A**) PERT availability in German hospital stratified by hospitals. (**B**) PERT availability in German hospital stratified by regions. (**C**) PERT activation in German hospital stratified by activation pathway. (**D**) Leading PERT in German hospital stratified by departments. (**E**) European PE reference centers versus German hospitals regarding PERT availability. (**F**) European PE reference centers versus German hospitals regarding availability PE follow-up programme

Overall, 65 hospitals participated at this German survey with most PERTs available at university hospitals (63%) and in West (63%) and Southern (62%) Germany (Fig. 1A and B). The most common method of activating PERTs are phone calls (71.1%), followed by mobile apps (23.7%) and pagers (5.2%) (Fig. 1C). In 86% cases, PERT is leading from the Department of Cardiology followed by Pulmonology (4.6%), Critical Care Medicine (2.3%) followed by Angiology (6.9%) (Fig. 1D). Regarding advanced treatment the majority of hospitals offers catheter-directed thrombolysis (72.2%) and catheter-based thrombectomy/aspiration (74.1%). Notably, almost half of the centers (49.6%) prioritize ST-elevation infarction over high-risk PE. Although 61.5% of the German centers handle over 40 PE cases (all risk clases) annually, only 52.3% of those hospitals already implemented a PERT, which is lower in comparison to the European Survey among 26 PE reference centers with more than 80% PERT implementation (Fig. 1E). A dedicated follow-up program for patients with PE is organized in 42.2% in German hospitals only. In the European PE reference centers almost all hospitals (92.3%) provide such a program (Fig. 1F).

## Discussion

Pulmonary embolism (PE), with its potentially fatal outcome, represents a major global health burden. In epidemiological studies, the incidence of PE has been reported to range between 39 and 115 cases per 100,000 population per year across Europe. Recent data from Germany indicate an incidence of approximately 99 cases per 100,000 population [1, 2] Before the development of PERT, the management of PE was often fragmented and inconsistent, leading to variable clinical outcomes, treatment delays, and underuse of advanced therapies such as catheter-directed thrombolysis or surgical embolectomy. The establishment of PERT introduced a coordinated, multidisciplinary approach designed to streamline diagnosis, risk stratification, and treatment decisions, ensuring timely and evidence-based optimized care for patients with acute PE.^3^ The potential positive impact of PERT on clinical outcomes remains a subject of ongoing discussion. Early studies demonstrated shifts in the utilization of advanced therapies when comparing the pre- and post-PERT eras [4]. For example, Rosovsky et al. reported that following PERT implementation, a larger proportion of patients underwent catheter-directed or other advanced interventions [5]. However, current evidence from randomized controlled trials suggests that interventional treatment in addition to anticoagulation may offer added benefit; therefore, the higher use of such interventions associated with PERT may contribute to improved outcomes in selected patients [6]. Additionally, subsequent studies have shown measurable improvements in several quality-of-care indicators after PERT implementation. These include reductions of in-hospital mortality and overall costs of care, suggesting that a structured, multidisciplinary response may promote more efficient and resource-conscious management of acute PE [7]. A meta-analysis observed similar trends and demonstrate a survival benefit among patients with pulmonary embolism [8]. The potential benefits of PERT on clinical outcomes remain inconclusive and require further research. Our data demonstrated a trend toward the management of PE in larger hospital centers with access to advanced interventions, particularly catheter-directed treatment. The increased use of such advanced therapies has previously been shown to coincide with the implementation of PERT [4, 8]. Regarding PERT leadership, the predominance of cardiologists—even when patients present via the emergency department - likely reflects established clinical workflows. In Germany, acutely unstable medical patients (e.g., in shock rooms or cardiac arrest) are often managed with cardiologist involvement as part of cardiogenic shock teams. Similarly, catheter-directed therapies are mainly performed by interventional cardiologists, although practices vary by center, with interventional radiologists involved in some hospitals. This heterogeneity reflects historical development and local organisational structures rather than formal national standardisation. Regardless of PERT lead, in general PERTs remain underrepresented in Germany compared with other European countries. Considering the reported potential benefits regarding cost reduction, shorter hospital stays, and improved clinical outcomes, there remains considerable room for improvement.

A not uncommon lack of digitalisation may also hamper and should be reflected in the activation process of PERTs, which in many cases still occurs primarily via telephone communication. Secure web-based platforms could improve care by enabling structured data sharing, multidisciplinary input, and better documentation. To understand and improve the treatment of PE a comprehensive and standardized follow-up program should be further developed and implemented in Germany. This survey demonstrated that among large German cardiovascular centers PERTs are markedly less often present compared to European PE reference centers. Additionally, in German centers, PE follow-up programs are not widely implemented. This findings emphasize the urgent need for awareness campaigns to enlighten physicians about importance of risk stratification, optimized timely treatment, mortality rates and long-term sequelae of PE.

## Data Availability

The datasets used and/or analysed during the current study are available from the corresponding author on reasonable request.

## Abbreviations

PE: Pulmonary embolism
PERT: Pulmonary embolism response team
